# 双配体磁性氢键有机框架材料的制备及用于糖基化肽与磷酸化肽的富集

**DOI:** 10.3724/SP.J.1123.2026.01031

**Published:** 2026-11-08

**Authors:** Yihan YANG, Chengyu CHENG, Yanjun LIU, Zhaoqing XIAO, Junwei YANG, Songrui LI, Haijiao ZHENG, Qiong JIA

**Affiliations:** 1. 吉林大学化学学院，吉林 长春 130012; 1. College of Chemistry，Jilin University，Changchun 130012，China; 2. 吉林大学国家级化学实验教学示范中心，吉林 长春 130012; 2. National Demonstration Center for Experimental Chemistry Education，Jilin University，Changchun 130012，China

**Keywords:** 氢键有机框架材料, 双配体, 磁性固相萃取, 质谱, 糖基化肽, 磷酸化肽, hydrogen-bonded organic frameworks, dual-ligand, magnetic solid-phase extraction（MSPE）, mass spectrometry （MS）, glycopeptides, phosphopeptides

## Abstract

蛋白质糖基化和磷酸化均是生命体中重要的翻译后修饰，它们影响着蛋白质的空间构象、生物活性、运输和定位，在细胞生长和分裂、信号转导、代谢途径等特定生物过程中发挥着至关重要的作用。本研究利用尿素配体中的氢原子与1，3，5-三（4-苯硼酸）苯配体中硼酸根的氧原子形成氢键，制备了一种双配体氢键有机框架材料（Fe_3_O_4_@HOF），并用于复杂实际样品中糖基化肽（糖肽）与磷酸化肽（磷肽）的富集。基于该双配体磁性HOF材料，建立了磁性固相萃取-质谱分析（MSPE-MS）平台，以标准蛋白酶解物为模型，考察了该方法对糖肽和磷肽的富集能力，检出限分别低至0.4和0.1 fmol/μL。结果表明，该方法在人唾液和血清样本中低丰度糖肽和磷肽的分析中展现出较好的选择性及分离能力，为高通量糖基化及磷酸化蛋白质组学分析提供了新的思路。

## Abstract (en)

Protein glycosylation and phosphorylation are critical post-translational modifications （PTMs） that regulate protein conformation，activity，transport，localization，and cellular processes including growth，division，signal transduction，and metabolism. However，the low abundance and poor ionization efficiency of glycosylated and phosphorylated peptides in complex biological samples necessitate highly selective enrichment strategies prior to mass spectrometry analysis. In this study，we designed and synthesized a dual-ligand magnetic hydrogen-bonded organic framework （Fe_3_O_4_@HOF） using 1，3，5-tris（4-phenylboronic acid）benzene and urea as complementary ligands. The resulting Fe_3_O_4_@HOF integrates the advantages of HOFs （well-defined structure，high specific surface area，and tailorable surface chemistry） with strong magnetic responsiveness （enabling rapid separation） and multiple synergistic enrichment mechanisms. The enrichment of glycopeptides is based on a cooperative mechanism combining boronate affinity （covalent binding between boronic acid and cis-diol groups of glycans） and hydrophilic interaction liquid chromatography （HILIC）. For phosphopeptide enrichment，electrostatic interaction between surface amino groups of the HOF and phosphate groups，together with HILIC effects，contribute to the highly efficient adsorption capacity. All enrichment experiments were performed using magnetic solid-phase extraction （MSPE） followed by matrix-assisted laser desorption/ionization time-of-flight mass spectrometry （MALDI-TOF MS） analysis. The analytical performance was evaluated using tryptic digests of standard glycoprotein （horseradish peroxidase，HRP） and phosphoprotein （α-casein）. The limit of detection （LOD） for glycopeptides was 0.4 fmol/μL，and for phosphopeptides was 0.1 fmol/μL，demonstrating the exceptional sensitivity. Selectivity was assessed by enriching target peptides from mixtures containing a 1 000-fold mass excess of bovine serum albumin. Under these challenging conditions，Fe_3_O_4_@HOF still enabled sensitive detection of both glycopeptides and phosphopeptides，confirming its outstanding specificity even in the presence of highly abundant interfering proteins. To validate practical applicability，the method was applied to human saliva and serum samples. From human saliva samples，Fe_3_O_4_@HOF successfully captured 29 distinct glycopeptides and 24 phosphopeptides，as identified by MALDI-TOF MS sequencing. From human serum samples，five typical endogenous phosphopeptides were unambiguously detected. These results confirm the reliability and accuracy of the method for real clinical samples and highlight its potential for large-scale PTM analysis in clinical proteomics，especially for profiling of glycosylation and phosphorylation. In summary，the dual-ligand magnetic HOF offers a powerful platform for high-specificity enrichment of both glycopeptides and phosphopeptides from complex biological matrices. Its excellent performance in clinical samples demonstrates its promise as a key tool for advancing clinical proteomics research，with potential applications in disease biomarker discovery and diagnostic support.

蛋白质翻译后修饰（post-translational modifications，PTMs）在众多细胞过程中发挥关键作用，如信号转导与调控过程、基因表达调控以及蛋白质相互作用等。因此，对蛋白质翻译后修饰的表征在细胞生物学以及疾病诊断与预防研究中至关重要。然而，由于翻译后修饰蛋白丰度较低、电离效率差，且大量非目标肽段会严重抑制信号，直接利用质谱技术分析翻译后修饰蛋白仍面临挑战^［1，2］^。目前已鉴定出400余种翻译后修饰类型，糖基化和磷酸化是最常见且重要的两种修饰。蛋白质糖基化通过增加蛋白质亲水性、影响局部构象或与分子相互作用，可帮助蛋白质形成正确的三维结构，指导蛋白质在细胞内的定向运输等。在免疫调节与疾病关联方面，糖基化模式影响抗体功能、炎症反应和自身免疫病发展，癌细胞表面糖基化异常与肿瘤转移、耐药性均相关，已发现的癌症标志物中糖蛋白占比较高^［3，4］^。另一种重要的PTM——蛋白质磷酸化是细胞生命活动的核心调控机制，其动态、可逆、快速的特性使其成为理想的信号开关。一些关键底物蛋白的磷酸化位点发生异常会导致细胞行为失控，引发癌症、神经退行性疾病等严重疾病^［5，6］^。针对磷酸化蛋白的检测是疾病诊断和预后判断的重要工具。不同的PTMs在调节蛋白质功能方面各司其职，并且许多蛋白质会同时发生两种或多种后修饰过程，这些共同修饰经常协同工作，形成复杂而精密的调控网络。例如，细胞表面受体需要糖链来确保其正确折叠并稳定定位于膜上，同时又需要磷酸化位点来快速传递胞外信号^［7］^。又如，正常脑脊液中的Tau蛋白是高度糖基化的，这有助于其稳定性和溶解性。在阿尔茨海默病病理中，Tau蛋白首先发生异常磷酸化，过度的磷酸化不仅使其失去功能，还可能触发或伴随其糖基化水平的下降，这种失衡促进了Tau蛋白的聚集和神经纤维缠结的形成^［8，9］^。可见，对同一生物样本中糖基化修饰和磷酸化修饰的同步监测是当前蛋白质组学研究的核心目标之一，目前尚无“一键式”的完美方案，需依赖多种技术的组合与策略创新。

基于质谱的后修饰组学是目前实现全局性、位点特异性同步分析的最有力工具之一。由于修饰蛋白在复杂生物样品中常被大量非修饰蛋白淹没，保证质谱分析结果准确性的关键在于样品预处理和富集策略。为了减少样本消耗、避免样本处理流程复杂化，开发能同时捕获糖基化肽（糖肽）与磷酸化肽（磷肽）的新型亲和材料势在必行。已开发的针对糖肽的富集方法有凝集素亲和法^［10，11］^、硼酸亲和法^［12-14］^和亲水相互作用色谱法（HILIC）^［15，16］^，对于磷肽，常使用固定金属离子亲和色谱法^［17，18］^、金属氧化物亲和色谱法^［19，20］^、静电亲和法^［21］^和亲水相互作用色谱法^［22］^等富集机理。不同的机理需要匹配合适的基质材料才能发挥最大效能，富集效率、选择性和重现性根本上取决于富集机理与基质材料的协同设计与优化。

能够精确控制键合密度和孔结构的多孔材料一直是糖肽和磷肽富集的优选基质^［23-25］^。与其他多孔材料（包括金属有机框架（MOFs）、无定形多孔有机材料（POPs）、共价有机框架（COFs）等）相比，氢键有机框架（HOFs）具有许多独特性质。与MOFs中的配位键、COFs中的共价键相比，HOFs中的氢键连接较弱，这可在结晶过程中引发可逆过程，从而产生高结晶度。这种高结晶度结构更有利于深入探索结构-功能关系。另一方面，HOFs的合成过程更为温和、简便。在结构多样性和可调性方面，HOFs可通过不同框架和氢键单元的组合来构建，因此，与非晶态聚合物有机框架相比，HOFs具有可调的孔径、孔形和孔结构^［26］^，且HOFs本身不含金属，其密度和毒性通常低于MOFs。在化学稳定性和热稳定性方面，尽管HOFs的稳定性不如由配位键和共价键构建的MOFs和COFs，但可以通过引入其他相互作用（如*π-π*堆积、范德华力等额外分子间作用力，或阴阳离子间的电荷作用等）实现HOFs稳定性的增强^［27］^。Brekalo等^［28］^报道了一种基于胍离子和芳香磺酸盐的双配体HOFs，该材料展现出优异的稳定性，这归因于其离子框架中存在的额外质子源。Xiong等^［29］^制备了一种基于盐酸胍和苯硼酸衍生物的双配体HOFs，该材料对单磷肽和糖肽展现出高效的富集效率，开拓了HOFs用于磷酸化修饰和糖基化修饰分析的应用。

本研究旨在构建一种双配体HOFs，用于糖肽与磷肽的富集，选取了可结合顺式二羟基的1，3，5-三（4-苯硼酸）苯（1，3，5-PBB）以及含有氨基和羰基的尿素分子作为该HOFs框架的双配体。在糖肽富集方面，该材料通过硼亲和作用与HILIC的协同效应，实现了对糖肽的高效捕获；对于磷肽，利用HOFs表面氨基与磷肽间的静电亲和作用，结合HILIC，达到了高效的富集效果。总体而言，所开发的双配体HOFs在富集并分析糖基化与磷酸化蛋白方面展现出巨大潜力，为HOFs在蛋白质组学分析中的应用提供了新途径。

## 1 实验部分

### 1.1 仪器、试剂与材料

JEM-2100F型场发射透射电镜（TEM，JEOL，日本）；Nicolet iS5型傅里叶变换红外光谱仪（FTIR，赛默飞世尔科技，美国）；SQUID-VSM型振动样品磁强计（VSM，Quantum Design Company，美国）；Empyrean型X射线衍射仪（XRD，PANalytical B.V.，荷兰）；Zetasizer Nano ZS ZEN3600型ZETA电位和纳米粒度分析仪（Malvern，英国）；AB Sciex 5800型基质辅助激光解析飞行时间质谱仪（MALDI-TOF MS，AB Sciex，美国）。

四水合氯化亚铁（FeCl_2_·4H_2_O，纯度≥98%）、六水合氯化铁（FeCl_3_·6H_2_O，纯度≥98%）、油酸（OA，纯度≥98%）、尿素（H_2_NCONH_2_，纯度99%）、1，3，5-PBB（纯度≥98%）、三氟乙酸（TFA，纯度≥98%）、甲酸（FA，纯度≥98%）、*N*-羟基丁二酰亚胺（NHS，纯度≥98%）、乙酸（HAc，纯度≥98%）、氨水（NH_3_·H_2_O，质量分数25%）、氯仿（CHCl_3_，纯度99%）、*N，N*-二甲基乙酰胺（DMA，纯度≥98%）、三乙胺（TEA，纯度≥98%）、1，2，4-三氯苯（1，2，4-TCB，纯度≥98%）均购自阿拉丁试剂（上海）。乙腈（ACN，纯度≥98%）、碳酸氢铵（NH_4_HCO_3_，纯度99%）、无水乙醇（EtOH，纯度99%）和磷酸（H_3_PO_4_，纯度≥98%）购自北京化工厂（北京）。胰蛋白酶（trypsin，纯度≥98%）、*α*-酪蛋白（*α*-casein，纯度99%）、辣根过氧化物酶（HRP，纯度99%）、1-（3-二甲氨基丙基）-3-乙基碳二亚胺盐酸盐（EDC，纯度≥98%）、牛血清白蛋白（BSA，纯度≥98%）和2，5-二羟基苯甲酸（DHB，纯度99%）均购自Sigma-Aldrich（美国密苏里州圣路易斯）。

### 1.2 实验方法

#### 1.2.1 样品前处理

标准样品处理：将1.0 mg HRP或*α*-酪蛋白溶解于1 mL NH_4_HCO_3_缓冲溶液（pH为8.2，50 mmol/L）中。另称取10 mg BSA，溶于含有8 mol/L尿素的1 mL NH_4_HCO_3_缓冲液中。在37 ℃条件下，以胰蛋白酶与蛋白质质量比为1∶40在样品中加入胰蛋白酶，孵育反应16 h，反应结束后，将制备的肽段溶液置于冰箱中冷冻备用。

实际样品处理：在100 μL唾液或血清样品中加入400 μL 40 mmol/L NH_4_HCO_3_缓冲溶液，于4 000 r/min、4 ℃的条件下离心16 min，取上层清液酶解后置于冰箱中冷冻备用，以备后续分析。

#### 1.2.2 Fe_3_O_4_@HOF的合成

图1为Fe_3_O_4_@HOF的合成路线图与其用于糖肽和磷肽的富集流程图。以二价铁与三价铁离子为前驱体（物质的量之比为1∶2），于N_2_氛围和碱性条件下反应。具体步骤如下^［29-32］^：在持续通入N_2_的条件下，将1.69 g的FeCl_3_·6H_2_O、1.24 g的FeCl_2_·4H_2_O与50 mL去离子水加入100 mL三颈烧瓶中混合。在剧烈搅拌过程中缓慢滴加7 mL NH_3_·H_2_O（质量分数为25%）。将反应体系升温至40 ℃维持30 min。反应结束后经真空干燥得到Fe_3_O_4_颗粒。向所得Fe_3_O_4_颗粒分散液中加入1 mL OA，于80 ℃下持续搅拌1 h，完成表面修饰步骤。使用无水乙醇对产物进行多次洗涤，真空干燥得到产物Fe_3_O_4_@OA。准确称取300 mg Fe_3_O_4_@OA与210 mg尿素依次溶于150 mL DMA溶液中，加入25 mg EDC，搅拌均匀，室温下反应2 h后加入36 mg NHS，随后室温下避光连续搅拌12 h，通过磁吸的方式收集得到黑色产物，依次用氯仿和去离子水洗涤产物3次，真空烘干，得到产物Fe_3_O_4_@Urea。随后，称取10 mg Fe_3_O_4_@Urea和20 mg 1，3，5-PBB加入7.5 mL TEA和6.0 mL 1，2，4-TCB的混合溶液中，超声混匀，将此溶液转移至预热至80 ℃的油浴中，在持续磁力搅拌下反应24 h。反应结束后，待反应体系冷却至室温，利用磁分离得到黑色磁性产物Fe_3_O_4_@HOF。为去除反应溶剂、过量的反应物及副产物，依次使用无水乙醇和去离子水对产物进行多次（至少3次）交替洗涤与磁分离操作。

**图1:**
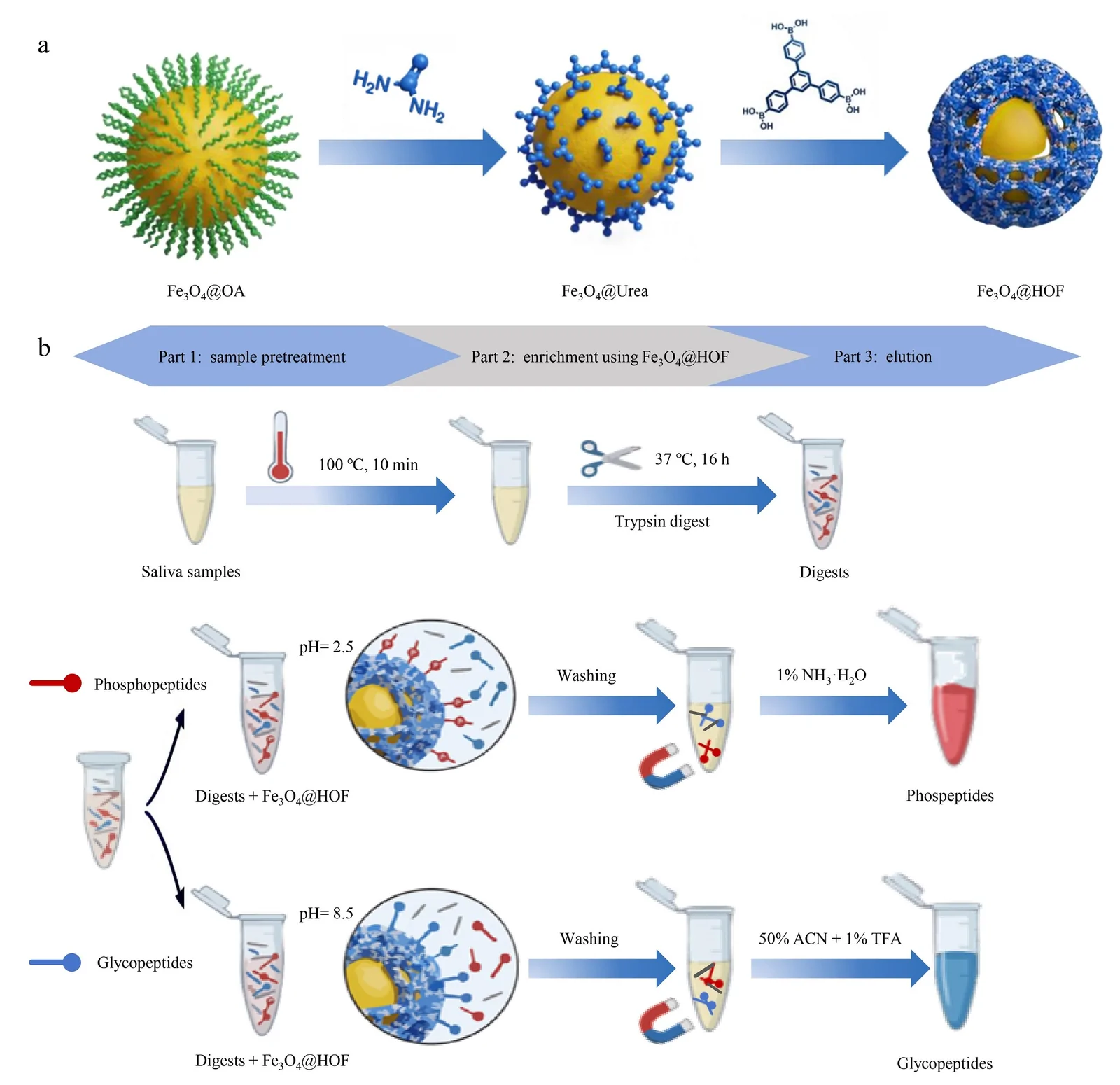
（a） Fe_3_O_4_@HOF的合成路线；（b） Fe_3_O_4_@HOF富集糖肽和磷肽的过程

#### 1.2.3 糖肽和磷肽的富集

取物质的量之比为1∶1的HRP与α-酪蛋白酶解物（稀释至浓度为1 μmol/L）于1 mL样品缓冲液（含0.1% TFA的90% ACN水溶液，pH为8.5）中，称取1 mg Fe_3_O_4_@HOF材料分散于该混合液中。首先富集糖肽，步骤如下：将该体系置于室温下孵育30 min，磁吸分离材料，保留该混合液并调节其pH至2.5。使用100 μL清洗缓冲液（含0.1% TFA的30% ACN水溶液）对材料进行反复清洗并进行磁分离，清洗后的材料置于30 μL洗脱液（含1% TFA的50% ACN水溶 液）中进行洗脱操作，收集糖肽的洗脱液。之后富集磷肽，步骤如下：将清洗后的材料重新置于保留的pH 2.5的混合液中，重复上述孵育、清洗与洗脱步骤，此过程中将洗脱液替换为NH_3_·H_2_O（质量分数为1%），收集洗脱液。以上两个过程所收集的洗脱液后续均使用MALDI-TOF MS 质谱仪进行测定。

#### 1.2.4 MALDI-TOF MS 的测试条件

选用反射正离子模式，扫描范围为*m/z* 1 000~3 500。基质溶液为加入40 mg DHB的40 mL含1% H_3_PO_4_的70% ACN水溶液。取0.5 µL基质溶液点于靶板上，再取0.5 µL的样品溶液覆盖靶板上的基质溶液。待靶板上的液滴结晶完全后，进行仪器测试。

## 2 结果与讨论

### 2.1 Fe_3_O_4_@HOF的表征

通过TEM图像分别观察Fe_3_O_4_和Fe_3_O_4_@HOF颗粒的内部结构和尺寸，见图2a和2b，Fe_3_O_4_和Fe_3_O_4_@HOF颗粒均呈现大小较均匀的球形形貌，Fe_3_O_4_颗粒尺寸较小，且团聚严重，而Fe_3_O_4_@HOF颗粒尺寸较大，表面粗糙，且颗粒分散性良好，这可能是因为HOF包覆在磁性颗粒表面形成一层多孔壳层，可阻止颗粒直接接触团聚。最后，结合动态光散射粒径分析（dynamic light scattering，DLS）结果可知，Fe_3_O_4_@HOF颗粒的粒径尺寸在约150~250 nm范围内，略大于Fe_3_O_4_颗粒的平均粒径尺寸。

**图2:**
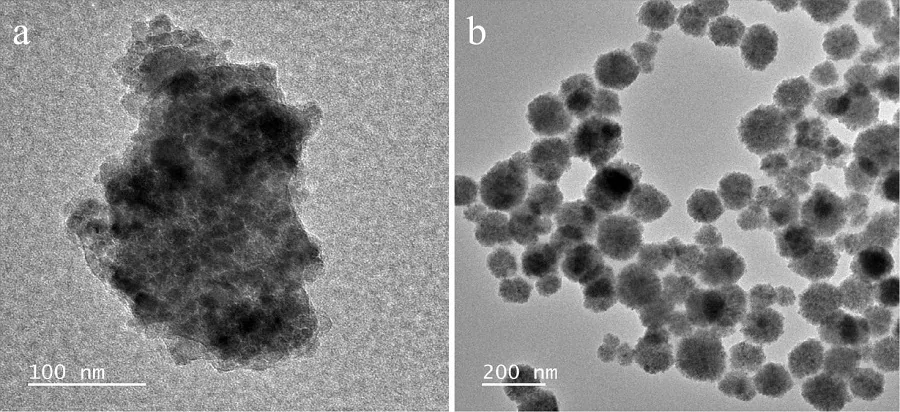
材料的透射电镜图像 a. Fe_3_O_4_； b. Fe_3_O_4_@HOF.

通过FTIR确定Fe_3_O_4_@HOF及中间产物的官能团和化学键信息，以验证Fe_3_O_4_@HOF的成功合成。由图3a可见，3种产物均存在Fe-O键和Fe-O-H键的特征吸收峰，其位置在576 cm^-1^和1 030 cm^-1^。对比中间产物Fe_3_O_4_@Urea，光谱图中出现归属于N-H键的伸缩振动和面内弯曲振动吸收峰，其位置在3 360 cm^-1^和1 620 cm^-1^，此外，还出现了归属于C-N键的伸缩振动峰，其位置在1 430 cm^-1^。以上信息可证明中间产物Fe_3_O_4_@Urea的成功合成。最后，对比Fe_3_O_4_@HOF材料的光谱图，在1 250 cm^-1^处出现B-O键吸收峰，在1 150 cm^-1^处出现B-Ar的吸收峰，说明苯硼酸基团的存在。此外，在3 300 cm^-1^处观察到宽而强的吸收带，归属于强氢键缔合的O-H伸缩振动。通过以上红外光谱信息初步验证了Fe_3_O_4_@HOF的合成。

**图3:**
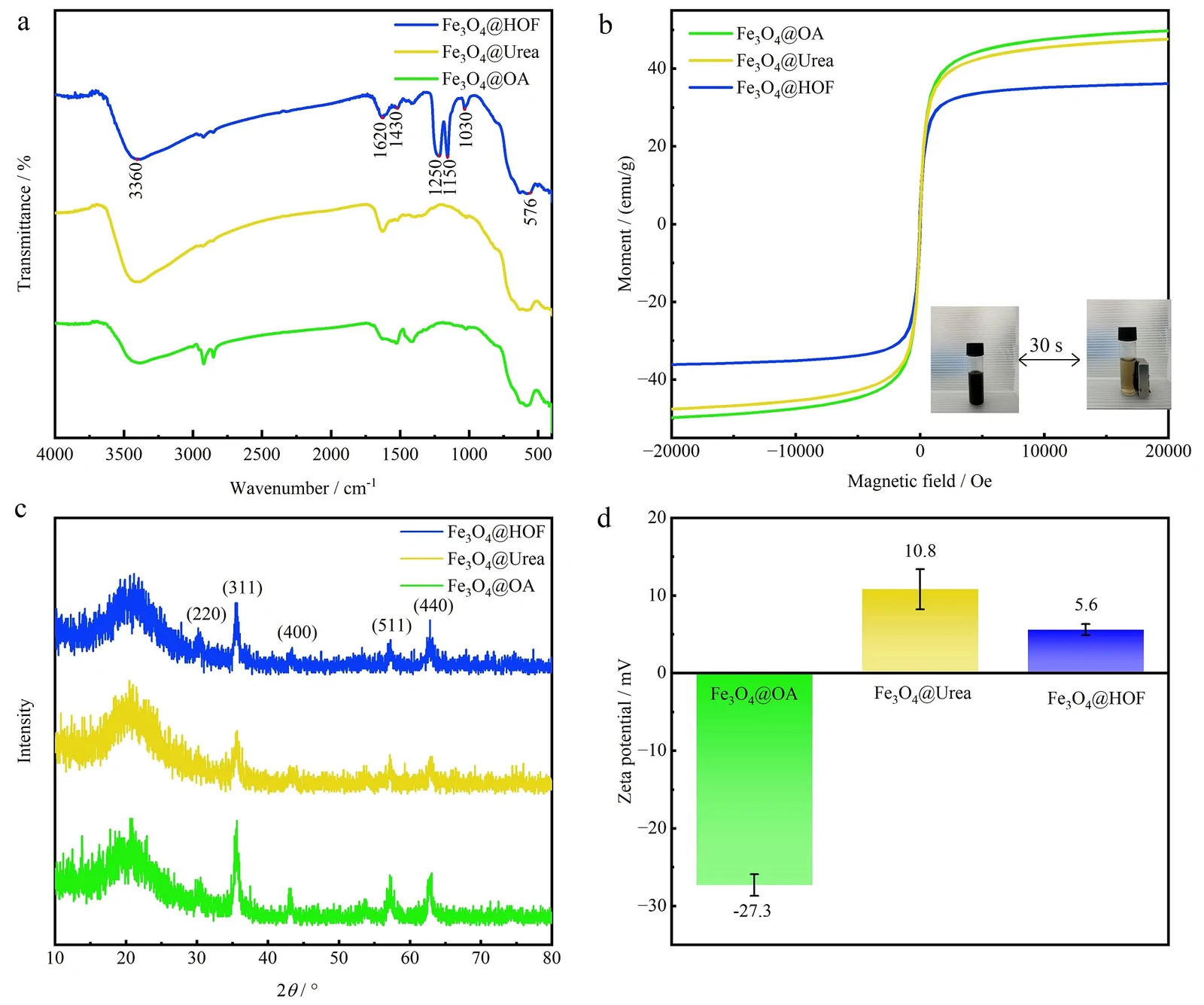
Fe_3_O_4_@OA，Fe_3_O_4_@Urea和Fe_3_O_4_@HOF的表征 a. FTIR spectra； b. vibrating sample magnetometer （VSM） magnetization curves； c. X-ray diffraction （XRD） patterns； d. zeta potentials （*n*=3）.

由图3b可见，所制备的Fe_3_O_4_@HOF及中间产物颗粒在室温下均表现出典型的铁磁性行为。对比3种产物，Fe_3_O_4_@HOF的饱和磁化强度（*Ms*）为36.0 emu/g，低于Fe_3_O_4_@OA（约49.5 emu/g）和Fe_3_O_4_@Urea（约47.3 emu/g），可归因于层层修饰扰乱了磁性颗粒的表面自旋，使参与集体磁化响应的有效磁矩减少，导致测得的*Ms*的下降。另一方面，VSM曲线在20 kOe的高场下已基本达到饱和，不存在高场下磁化强度持续线性增加的情况。结合图3c的XRD谱图信息，与标准卡片（JCPDS No. 19-0629）对比，所有衍射峰均与立方尖晶石结构的Fe_3_O_4_完美匹配，无杂相峰出现。以上表征信息均证明所合成的材料具有较好的Fe_3_O_4_晶体结构，且磁响应性良好，在30 s内可实现材料与溶剂的磁分离（见图3b内插图），满足磁性吸附剂快速分离的要求。

由图3d可见，所制备的Fe_3_O_4_@HOF及中间产物的表面电荷呈现出较大的差异性。Fe_3_O_4_@OA颗粒的Zeta电位为（-27.3±2.4） mV，该负电荷主要来源于油酸壳层表面羧基的去质子化，且绝对值近似30 mV，表明该材料具有优异的稳定性。与Fe_3_O_4_@OA相比，Fe_3_O_4_@Urea的Zeta电位（约（10.8±4.9） mV）显著向正值移动，说明在水中尿素表面的氨基结合质子形成了带正电的铵根离子。将1，3，5-PBB接枝到Fe_3_O_4_@Urea上得到Fe_3_O_4_@HOF后，其Zeta电位小幅下降至约（5.6±0.9） mV，其主要原因为疏水性的1，3，5-PBB层覆盖了Fe_3_O_4_@Urea表面带正电的铵根离子。同时，该疏水层也导致颗粒在分散中表现出轻微的聚集倾向，这解释了其Zeta电位分布略微宽化的现象。

### 2.2 Fe_3_O_4_@HOF材料的理论模拟计算

在HOF的合成过程中，双配体1，3，5-PBB和尿素通过氢键和配位键组装成框架。每个硼酸阴离子通过氢键与氨基相连，而每个尿素分子连接两个硼酸阴离子。图4显示了3种不同构象的1，3，5-PBB（图4a~c）和尿素分子（图4d）与典型的单糖单元*N*-乙酰神经氨酸（*N*-acetylneuraminic acid，Neu5Ac）之间的结合模式。如表1所示，不同构象的1，3，5-PBB与糖分子之间均具有良好的结合能力，其中前两种构象的1，3，5-PBB与糖分子之间具有非常强的结合能力。尿素与糖分子之间结合能力较弱，但仍提供了氢键作用。图4e和4f表明尿素与典型的单磷单元（1-丁基磷酸）之间存在静电相互作用，同时，静电势计算亦显示出尿素与Neu5Ac之间存在静电互补性，表明本研究所构建的HOF有利于对糖肽和磷肽的选择性富集^［33］^。

**图4:**
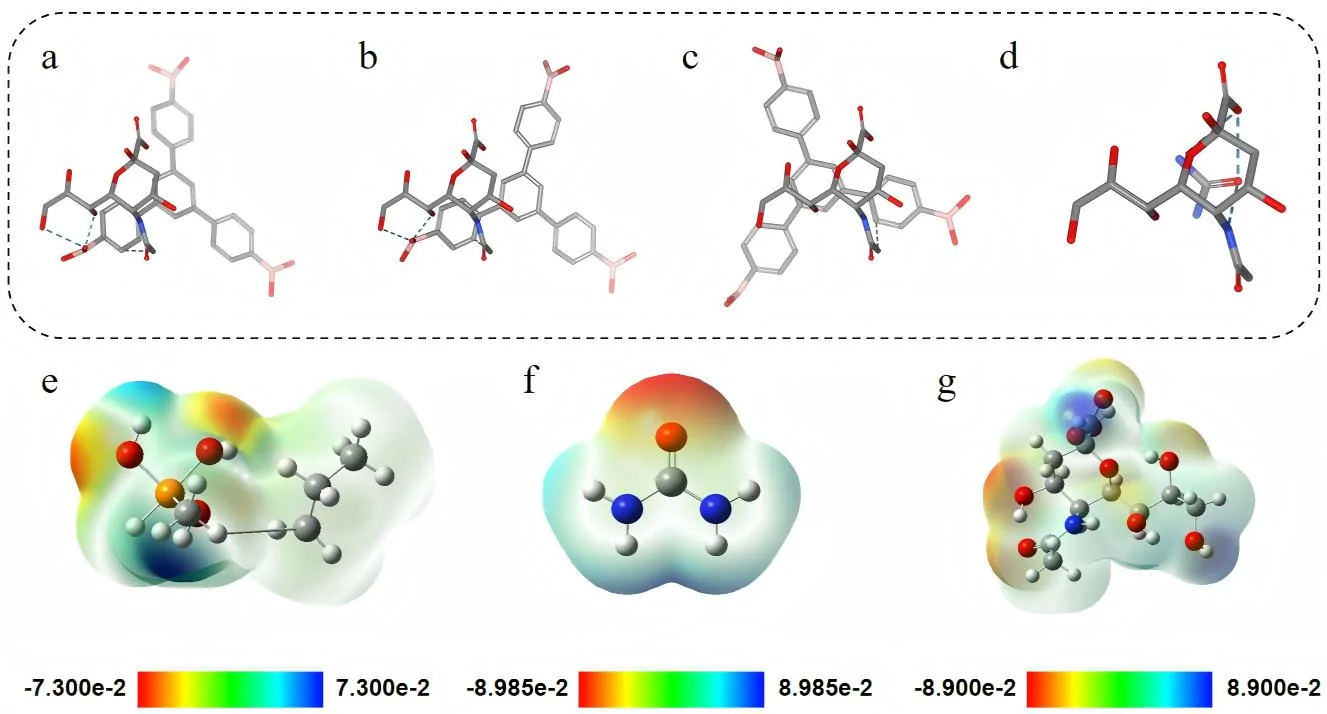
理论模拟计算 Model diagrams of the interaction of （a，b，c） 1，3，5-tris（4-phenylboronic acid）benzene （1，3，5-PBB） and （d） urea with *N*-acetylneuraminic acid （Neu5Ac）； molecular electrostatic potential maps of （e） 1-butyl phosphate，（f） urea，and （g） Neu5Ac.

**表1:** 3种构象的1，3，5-PBB与尿素分子分别与Neu5Ac之间的结合能力打分

Conformation	Configuration	Grid score	Grid vdw energy	Grid es energy	Internal repulsive energy
C_24_H_21_B_3_O_6_	1	-100.068481	24.771654	-124.840134	95.606689
C_24_H_21_B_3_O_6_	2	-58.086761	19.797279	-77.884041	64.795837
C_24_H_21_B_3_O_6_	3	-12.761647	-12.555444	-0.206204	70.14386
H_2_NCONH_2_	1	-5.8718	-5.677217	-0.194583	0.000003

### 2.3 Fe_3_O_4_@HOF对糖肽和磷肽富集能力的评估

以酶解后的HRP和*α*-酪蛋白为标准样品，考察Fe_3_O_4_@HOF对糖肽和磷肽的富集能力。由于本实验针对糖肽和磷肽的富集机理为硼亲和作用、静电作用和亲水相互作用，所以优化缓冲液的pH和极性条件十分重要。对于富集糖肽的硼亲和机理而言，硼酸基团在碱性水溶液中主要以带负电的四面体硼酸阴离子形式存在，这使其能够与糖分子发生快速、可逆的共价结合，在酸性或中性条件下，硼酸主要以电中性的平面三角形B（OH）_3_形式存在，与糖分子的结合能力很弱，易被洗脱液洗脱。对于HILIC，有机相比例越高，流动相洗脱能力越弱，分析物保留越强。大多数极性化合物在70%~95%的ACN水溶液中均有良好的保留和峰形。硼亲和法需要足够的水相来维持缓冲液pH为碱性。本研究通过控制单一变量法确定最佳实验参数组合，选取洗脱液为1% TFA的50% ACN水溶液，以5%的增量微调样品缓冲液的有机相比例，观察典型糖肽的丰度变化以确定最佳的缓冲液条件。由图5a可见，选取*m*/*z* 2 398.1、2 852.5和3 672.8 3个典型的糖肽特征峰作为参照，当样品缓冲液pH为8.5时，90% ACN水溶液中典型糖肽特征峰的丰度最高。这表明，在此条件下，HILIC与硼亲和富集机理协同作用，电中性的硼酸基团失去质子，转化为带负电且反应活性更高的硼酸盐形式，从而实现了糖肽的高效富集。

**图5:**
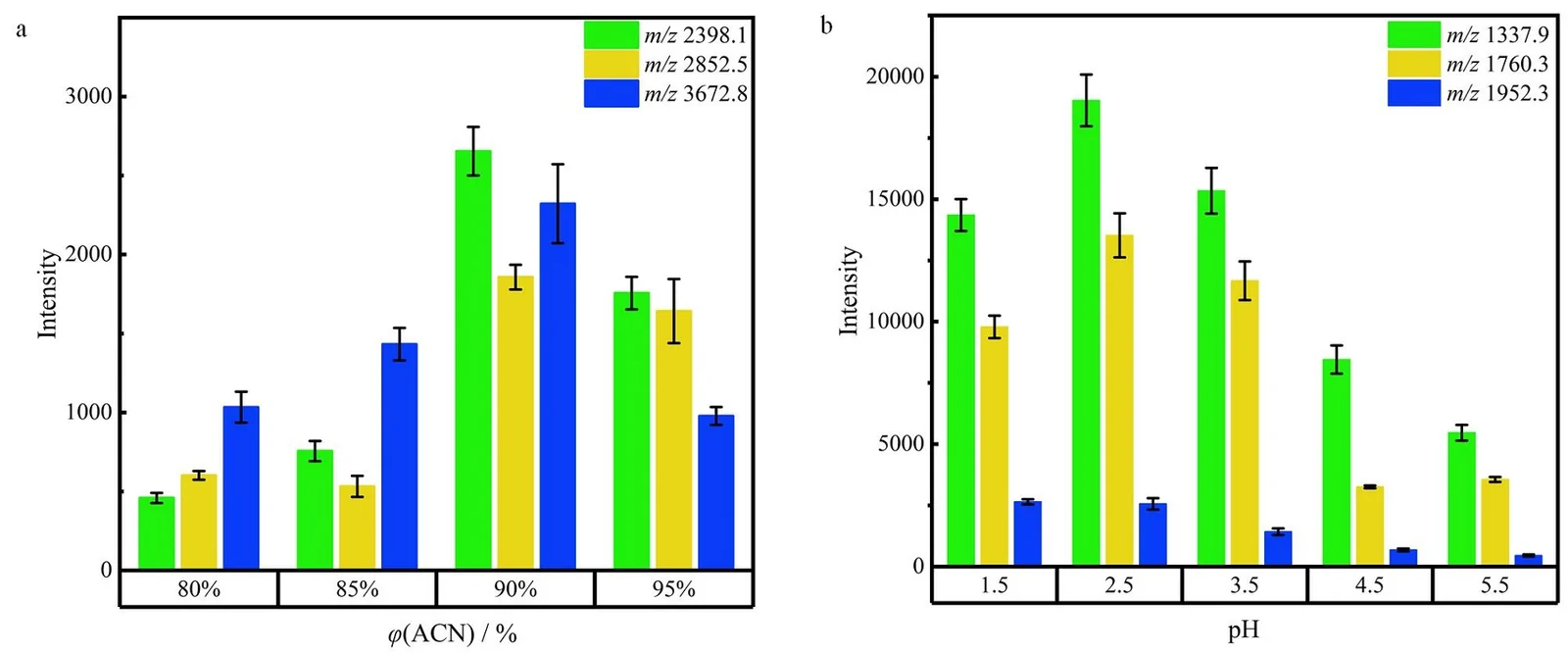
缓冲溶液中乙腈体积分数和pH对富集能力的影响（*n*=3**）** a. HRP； b. *α*-casein.

同样地，对于基于静电相互作用和亲水相互作用富集磷肽的方法，pH决定了磷酸根和氨基的电离状态，从而决定了材料对磷肽的结合能力^［34，35］^。固定洗脱液为NH_3_·H_2_O（质量分数为1%），优化样品缓冲液的pH范围为1.5~5.5；选取*m*/*z* 1 337.9、1 760.3和1 952.3的3个典型的磷肽特征峰作为参照，结果见图5b。实验结果表明，当样品缓冲液pH为2.5时，谱图中的磷肽特征峰最为明显，且背景干净；选择该pH的原因是，在此条件下磷酸基团的部分去质子化使其带负电，同时降低非目标酸性肽段的干扰，实现了磷肽的高选择性富集。

在最佳的实验条件下，选取浓度为4.0 fmol/μL的标准蛋白溶液，比较富集前后糖肽和磷肽的质谱信号。图6a和6d为未经富集的质谱图，可见谱图中无目标肽段的信号，在经Fe_3_O_4_@HOF富集后，谱图中出现明显的12条糖肽（图6b）和9条磷肽（图6e）的信号峰，所富集到的糖肽和磷肽信息见表2。在最佳实验条件下考察方法的灵敏度，先将酶解后的HRP和*α*-酪蛋白溶液分别稀释至40 fmol/μL，通过MALDI-TOF MS进行检测，当观察到谱图中的目标肽段峰信噪比（*S*/*N*）远高于3时，再次稀释样品溶液至更低浓度。结果如图6所示，本方法在稀释至0.4 fmol/μL的样品中依然可以检测到4条*S*/*N>*3的糖肽（图6c），在稀释至0.1 fmol/μL的样品中可以检测到5条*S*/*N>*3的磷肽（图6f），与近年其他文献所报道的方法进行对比（表3和表4），本方法对糖肽和磷肽具有较高的灵敏度。

**图6:**
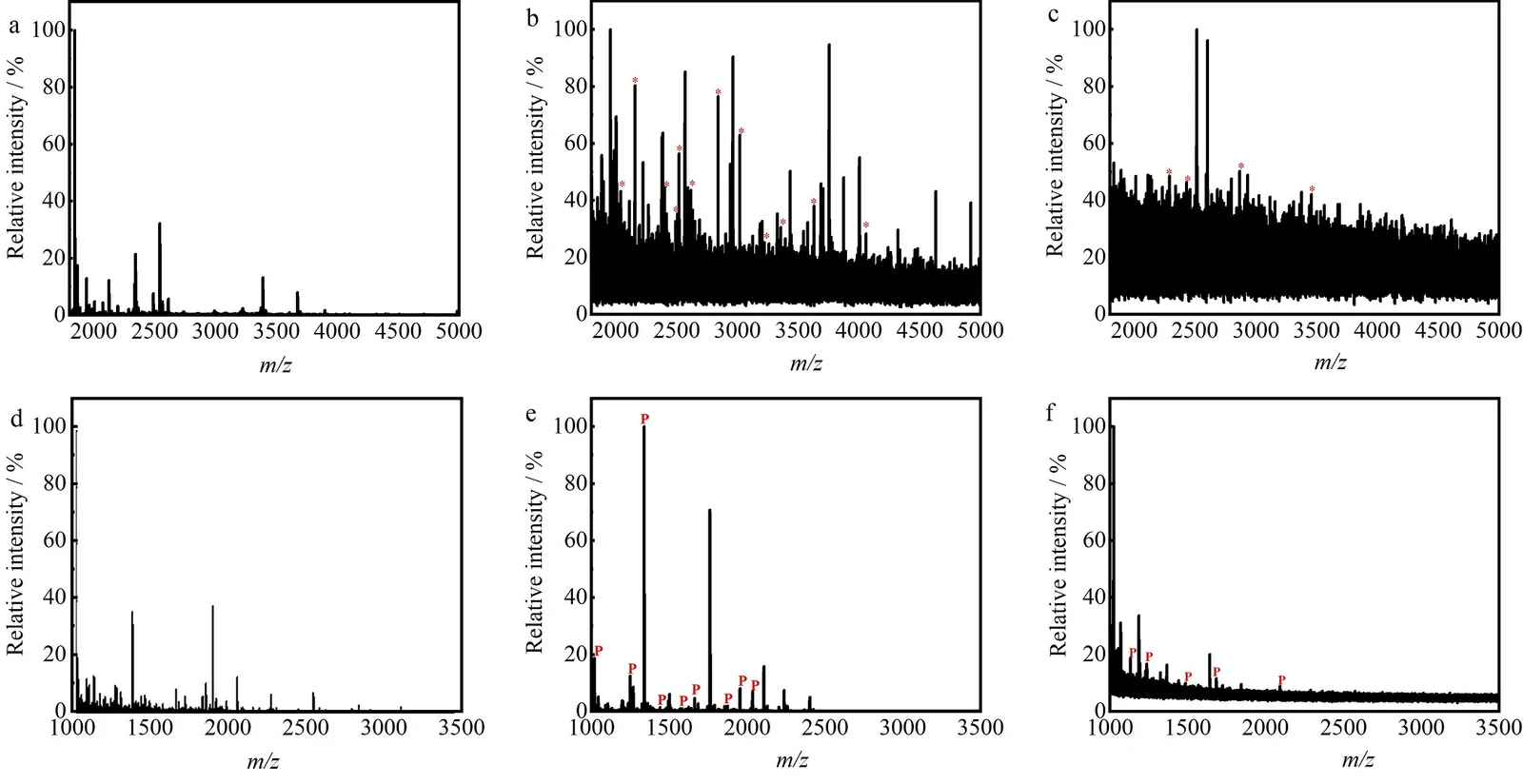
经Fe_3_O_4_@HOF富集后HRP和 ***α*** -酪蛋白酶解液的MALDI-TOF质谱图 a，d. direct analysis； b，e. 40 fmol/μL； c. 0.4 fmol/μL； f. 0.1 fmol/μL. * represents glycopeptide. P represents phosphopeptide.

**表2:** HRP和 ***α*** -酪蛋白酶解液中的糖肽和磷肽

Peak No.	Observed *m/z*	Peptide sequence （glycoform）	Residue
1	1843.42	NVGLN#R （XylMan3FucGlcNAc2）	P00433/184-189
2	2150.01	LYN#FSN#TGLP （Man3GlcNAc2Xyl）	P00433/214-223
3	2398.22	N#LEEQKGLI （Hex3HexNAc3Fuc1）	P00433/266-274
4	2488.44	N#LEEQKGLI （Hex3HexNAc4）	P00433/266-274
5	2542.76	SSPN#ATDTIPLVR （XylMan3GlcNAc2Fuc）	P00433/282-294
6	2612.34	MGN#ITPLTGTQGQIR （XylMan3GlcNAc2）	P00433/304-328
7	2852.69	GLIQSDQELFSSPN#ATDTIPLVR （GlcNAcFuc）	P00433/272-294
8	3046.58	SFAN#STQTFFNAFVEAMDR （XylMan2GlcNAc2）	P00433/181-213
9	3257.45	SFAN#STQTFFNAFVEAMDR （XylMan3FucGlcNAc2）	P00433/181-213
10	3389.31	DSFRNVGLN#R （XylHex6HexNAc4Fuc2）	P00433/180-189
11	3672.48	GLIQSDQELFSSPN#ATDTIPLVR （XylMan3GlcNAc2Fuc）	P00433/272-294
12	3895.99	LHFHDCFVNGCDASILLDN#TTSFR （XylMan3GlcNAc2Fuc）	P00433/69-92
13	1031.32^*^	FQsEEQQQTEDELQDK	P02666/48-63
14	1237.47^*^	TVDMEsTEVF	P02663/153-162
15	1337.28^*^	HVsSSEESII	P02662/21-30
16	1466.83^*^	TVDMEsTEVFTK	P02663/153-164
17	1564.21^*^	RELEELNVPGEIVEsLsssEESITRI	P02666/16-41
18	1660.27^*^	VPQLEIVPNsAEER	P02662/121-134
19	1863.58^*^	DIGSEsTEDQAmEDIK	P02662/58-73
20	1951.74^*^	YKVPQLEIVPNsAEER	P02662/119-134
21	2061.82^*^	FQsEEQQQTEDELQDK	P02666/48-63

* represents phosphopeptide.

**表3:** 不同材料对糖肽富集能力的对比

Material	LOD/（fmol/μL）	Selectivity	Ref.
NUS-10@CS	1.0	HRP∶BSA （1∶2000，*m*/*m*）	［^36^］
FZT-GSH	0.1	HRP∶BSA （1∶50，*m*/*m*）	［^37^］
MPDA@PEI@CA	5.0	HRP∶BSA （1∶5000，*m*/*m*）	［^38^］
Fe_3_O_4_@TpBD@Au@GSH	0.1	HRP∶BSA （1∶2000，*m*/*m*）	［^39^］
MUiO-66-NH_2_/PA	1.0	HRP∶BSA （1∶1000，*m*/*m*）	［^40^］
Fe_3_O_4_@ILI-01@Ti^4+^	0.1	HRP∶BSA （1∶500，*m*/*m*）	［^41^］
Fe_3_O_4_@HOF	0.4	HRP∶BSA （1∶1000，*m*/*m*）	this work

**表4:** 不同材料对磷肽富集能力的对比

Material	LOD/（fmol/μL）	Selectivity	Ref.
ZrP-OTCC	10	*α*-casein∶BSA （1∶100，*m*/*m*）	［^42^］
SiO_2_-La_2_O_3_	50	*α*-casein∶BSA （1∶100，*m*/*m*）	［^43^］
Fe_3_O_4_@SiO_2_@（Zr-Ti-MOF）_10_-NH_2_	1.0	*β*-casein∶BSA （1∶2000，*m*/*m*）	［^44^］
Fe_3_O_4_@TCPP-DOTA-Tb^3+^	100	*α*-casein∶BSA （1∶50，*m*/*m*）	［^45^］
TpPa-2-Ti^4+^	4.0	*α*-casein∶BSA （1∶50，*m*/*m*）	［^46^］
Fe_3_O_4_@ZW-MOF	1.0	*β*-casein∶BSA （1∶1000，*m*/*m*）	［^47^］
Fe_3_O_4_@HOF	0.1	*α*-casein∶BSA （1∶1000，*m*/*m*）	this work

为考察该方法的选择性，将酶解后的HRP和*α*-酪蛋白与酶解后的BSA按照1∶1∶1000的质量比混合。结果如图7所示，MALDI-TOF MS检测结果表明，对复杂样品进行直接检测时无法检测到糖肽和磷肽的质谱峰（图7a）；经Fe_3_O_4_@HOF富集后，非目标肽段的峰被显著抑制，可观察到较强的糖肽和磷肽信号峰（图7b和7c）。

**图7:**
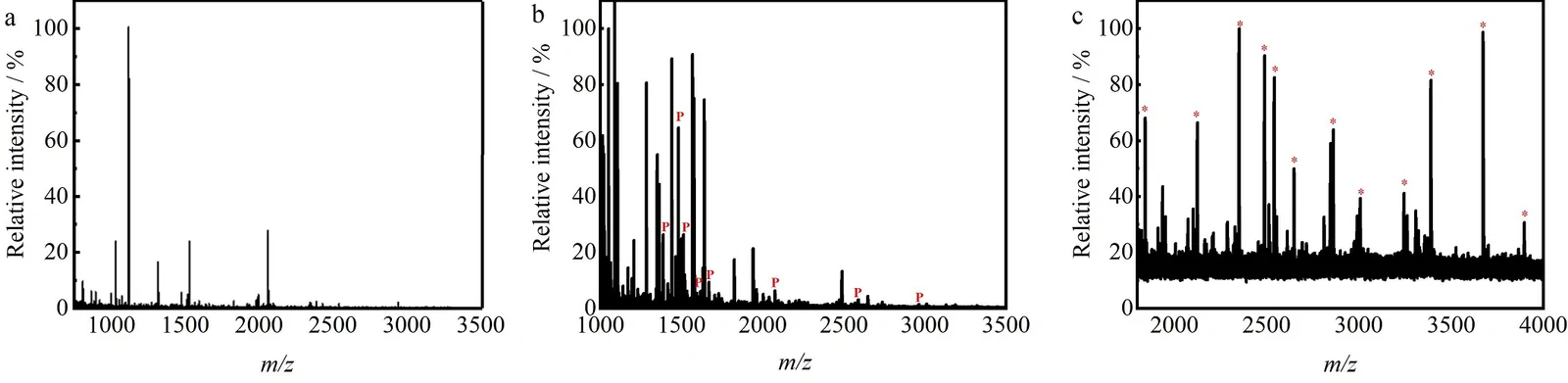
HRP和 ***α*** -酪蛋白与BSA混合样品酶解液的MALDI-TOF质谱图 a. direct analysis； b，c. analysis after enrichment by Fe_3_O_4_@HOF. * represents glycopeptide. P represents phosphopeptide.

### 2.4 实际样品分析

为了全面且精准地评估Fe_3_O_4_@HOF在复杂生物基质中对低丰度糖肽和磷肽的捕获能力，本研究以公认的疾病生物标志物重要来源——人唾液和血清作为实际生物样本展开实验探究，相关实验结果呈现于图8。

**图8:**
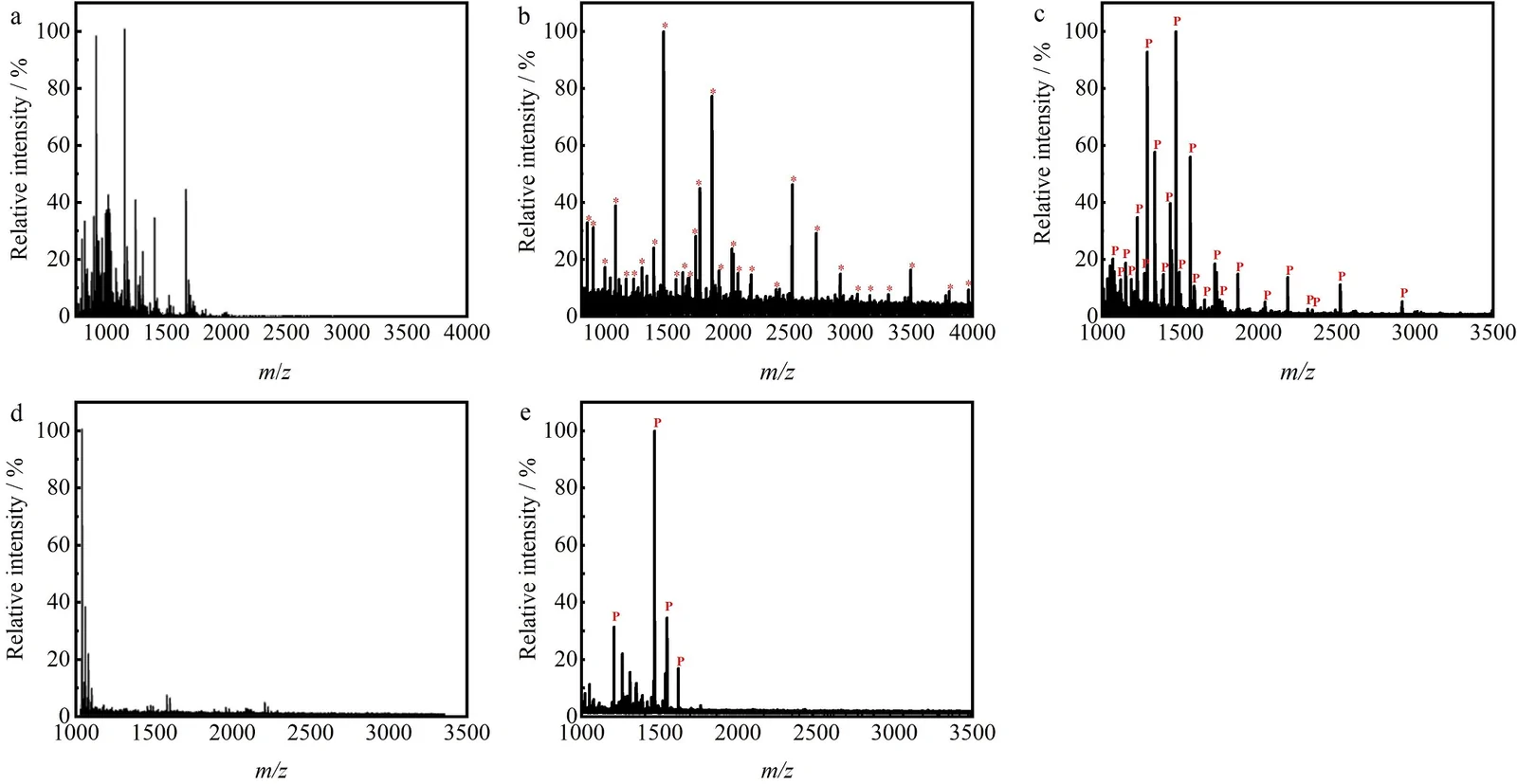
（a，b，c）唾液样品和（d，e）血清样品的MALDI-TOF质谱图 a，d. direct analysis； b，c，e. analysis after enrichment by Fe_3_O_4_@HOF. * represents glycopeptide. P represents phosphopeptide.

在未使用Fe_3_O_4_@HOF进行富集处理时，从质谱图中可以清晰观察到，由于人唾液样品中存在大量非目标肽段，同时基质干扰情况十分严重，糖肽和磷肽的信号极其微弱，几乎难以被有效观测到（图8a和8d）。这一现象充分表明，在复杂生物样本中，直接检测低丰度的糖肽和磷肽面临着巨大的挑战。而当运用Fe_3_O_4_@HOF对样品进行富集处理后，情况发生了显著变化。在人唾液样品中，成功检测到了29条糖肽（图8b）和24条磷肽（图8c）。进一步分析发现，这些检测到的糖肽大多归属于碱性唾液富脯氨酸蛋白4（basic salivary proline-rich protein 4）、*α*-2-HS糖蛋白（*α*-2-HS-glycoprotein）、抗凝血酶-Ⅲ（antithrombin-Ⅲ）等典型的会发生糖基化修饰的蛋白质；而磷肽则归属于唾液酸性富脯氨酸磷酸蛋白1/2（salivary acidic proline-rich phosphoprotein 1/2）、富酪蛋白（statherin）、碱性唾液富脯氨酸蛋白2（basic salivary proline-rich protein 2）等会发生磷酸化修饰的蛋白质。人唾液样品中检测到的糖肽的详细信息列于表5。

**表5:** 唾液样品经Fe_3_O_4_@HOF材料富集处理后得到的糖肽信息

Peak No.	Observed *m/z*	Peptide sequence	Residue
1	826.62	AIN#DTAAR	A0A3B3ISU0/484-491
2	972.56	INGN#cTGIK	P63096/201-209
3	990.58	N#NSDISSTR	P01871/46-54
4	1076.59	KEDALN#ETR	P10909/81-89
5	1165.61	GPPPQEGN#KSR	P10163/227-237
6	1225.75	EN#ISDPTSPLR	P01591/70-80
7	1333.73	N#LTTSLTESVDR	P80108/307-318
8	1399.83	VPGN#VTAVLGETLK	P01833/466-479
9	1471.78	VYKPSAGN#NSLYR	P02749/155-167
10	1572.88	N#HScEPcQTLAVR	P00740/46-58
11	1627.90	PGKPQGPPPQGGN#QSQG	P10163/54-70
12	1680.99	KPEGPPPQEGN#KSRSA	P10163/224-239
13	1732.94	ALPQPQN#VTSLLGcTH	P02790/447-462
14	1771.97	VcQDcPLLAPLN#DTR	P02765/145-159
15	1866.99	LGAcN#DTLQQLMEVFK	P01008/124-139
16	1924.12	PGKPEGPPPQGGN#QSQGPPP	Q04118/54-73
17	2021.10	IPcSQPPQIEHGTIN#SSR	P08603/868-885
18	2078.15	AFGQFFSPGEVIYN#KTDR	Q9HC84/4947-4964
19	2188.15	LGSFEGLVN#LTFIHLQHNR	P51884/152-170
20	2345.13	SQILEGLGFN#LTELSESDVHR	P29622/98-119
21	2522.34	QIGLYPVLVIDSSGYVNPN#YTGR	P01833/168-190
22	2678.43	MVSHHN#LTTGATLINEQWLLTTAK	A0A0C4DGL8/120-143
23	2722.47	ADGTVNQIEGEATPVN#LTEPAKLEVK	P00533/302-327
24	2916.53	NVVDGQPFTNWYDN#GSNQVAFGRGNR	P05067/686-711
25	3160.45	QGPPPQGGNQPQGPPPPGKPQGPPPQGGsKS	P10163/58-88
26	3312.75	PPPPGKPQGPPPQGGN#QSQGPPPPGKPQGPPPQGG	P10163/51-86
27	3380.53	QNLNEDVSQEESPSLIAGN#PQGAPPQGGNKPQ	P02812/17-48
28	3578.72	PPGKPQGPPPQGGN#QSQGPPPPGKPQGPPPQGGsKS	P10163/53-88
29	3964.91	FVQAIcEGDDcQPPAYTYN#NITcASPPEVVGLDLR	P35858/571-595

类似地，在人血清样品中，经过Fe_3_O_4_@HOF材料的富集处理后，共检测到5条磷肽（图8e），且这些磷肽全部归属于人血清中典型的Fibrinogen A（FGA）磷蛋白。人唾液和血清样品中所检测到的磷肽的详细信息均列于表6，为全面了解磷肽在人体不同生物样本中的分布情况提供了依据。

**表6:** 唾液和血清样品经Fe_3_O_4_@HOF材料富集处理后得到的磷肽信息

Peak No.	Observed *m/z*	Peptide sequence	Residue
1	1075.48	SsEEKFLR	P02808/21-28
2	1155.44	ssEEKFLR	P02808/21-28
3	1190.51	DsSEEKFLR	P02808/20-28
4	1232.48	sEQFIDEER	P02810/38-46
5	1270.47	DssEEKFLR	P02808/20-28
6	1293.59	DVsQEDVPLVI	P02812/25-32
7	1311.54	ssEEKFLRR	P02808/21-29
8	1360.54	sEQFIDEERQ	P02810/38-47
9	1382.57	DVPLVISDGGDsE	P02810/27-37
10	1426.57	DssEEKFLRR	P02808/20-29
11	1461.55	GGDsEQFIDEER	P02810/35-46
12	1494.60	sGVPGQPGsPGLPGQ	P15515/51-56
13	1576.58	DGGDsEQFIDEER	P02810/34-46
14	1589.61	GGDsEQFIDEERQ	P02810/35-47
15	1647.64	GAPGAPGPKGEsGDyK	P02746/102-117
16	1712.81	PGKPQGPPPQGDKSRs	P10163/272-288
17	1742.80	GPAGPPGPPGPPGPPGVsGGG	P08123/1087-1107
18	1875.76	VISDGGDsEQFIDEER	P02810/31-46
19	2020.76	DVsQEDVPLVISDGGDsE	P02810/22-37
20	2182.09	GsRPGDARPAGAPGAAARVLTPG	F8W883/1391-1412
21	2284.12	PGKPQGPPPQGGNKsQGPPPPGK	P02812/242-262
22	2334.00	GAPGPAGsRGAPGPQGPRGDKGEt	P02461/1074-1097
23	2508.02	DVPLVIsDGGDsEQFIDEERQ	P02810/27-46
24	2920.44	TFsWEEIQKHNLRTDRWLVIDR	O95864/20-41
25	1309.6^*^	DsGEGDFLAEGGGV	P02671/2-15
26	1465.7^*^	ADsGEGDFLAEGGGV	P02671/2-16
27	1537.7^*^	ADsGEGDFLAEGGGVR	P02671/1-16
28	1545.6^*^	DsGEGDFLAEGGGVR	P02671/2-16+P
29	1616.7^*^	ADsGEGDFLAEGGGVR	P02671/1-16+P

* represents serum phosphopeptide.

## 3 结论

本研究合成了一种基于1，3，5-三（4-苯硼酸）苯与尿素双配体的双功能磁性氢键有机框架材料Fe_3_O_4_@HOF，其结构明确性、高比表面积以及表面化学的精确可设计性，为糖肽与磷肽的富集提供了理想平台。该材料充分利用了HOFs固有的结构优势、多重协同的富集机理以及材料的高磁响应性，实现了对复杂生物样品中糖肽与磷肽的高特异性分离与富集。该HOFs具有以下特点：1）借助尿素单体中的氨基与磷酸根形成静电亲和作用，该材料可实现磷肽的有效富集；2）利用苯硼酸衍生物配体与糖分子中的顺式二羟基之间的硼亲和作用实现糖肽的富集；3）尿素配体赋予HOFs较强的亲水性，利用HILIC增强对糖肽和磷肽的双富集能力；4）HOFs内部氢键网络进一步强化了其对糖肽与磷肽的亲和力；5）该HOFs具有四氧化三铁磁性颗粒所带来的磁性，简化了富集操作流程。结合MALDI-TOF MS这一高灵敏度检测技术，Fe_3_O_4_@HOF展现出了对糖肽（灵敏度低至0.4 fmol/μL）和磷肽（灵敏度低至0.1 fmol/μL）的卓越检测性能，同时，在目标肽段与BSA混合样品的检测中表现出了优异的选择性。随后，将Fe_3_O_4_@HOF应用于唾液和血清等实际临床样品的分析，结果显示，该材料能够成功捕获人唾液样品中的29条糖肽和24条磷肽，以及人血清样品中的5条典型磷肽，这一成果不仅验证了本方法在临床样品中糖肽和磷肽分析的可靠性与准确性，更凸显了其在临床蛋白质组学研究，尤其是规模化糖基化和磷酸化修饰分析中的巨大应用潜力。展望未来，基于双配体HOFs的多孔框架材料有望成为推动临床蛋白质组学研究向更高灵敏度、更高选择性方向发展的关键工具，为疾病标志物的发现与临床诊断提供强有力的技术支持。
